# Aerobic Respiration and Its Regulation in the Metal Reducer *Shewanella oneidensis*

**DOI:** 10.3389/fmicb.2021.723835

**Published:** 2021-09-09

**Authors:** Kristen Bertling, Areen Banerjee, Daad Saffarini

**Affiliations:** Department of Biological Sciences, University of Wisconsin-Milwaukee, Milwaukee, WI, United States

**Keywords:** aerobic respiration, *Shewanella oneidensis* MR1, crp, cAMP phosphodiesterase, anti-sigma factor, cytochrome oxidase

## Abstract

*Shewanella oneidensis* MR-1 is a facultative anaerobe known for its ability to reduce metal oxides. Anaerobic respiration, especially metal reduction, has been the subject of extensive research. In contrast, *S. oneidensis* aerobic respiration has received less attention. *S. oneidensis* expresses *cbb_3_*- and *aa_3_*-type cytochrome *c* oxidases and a *bd*-type quinol oxidase. The *aa_3_*-type oxidase, which in other bacteria is the major oxygen reductase under oxygen replete conditions, does not appear to contribute to aerobic respiration and growth in *S. oneidensis*. Our results indicated that although the *aa3*-type oxidase does not play a role in aerobic growth on lactate, the preferred carbon source for *S. oneidensis*, it is involved in growth on pyruvate or acetate. These results highlight the importance of testing multiple carbon and energy sources when attempting to identify enzyme activities and mutant phenotypes. Several regulatory proteins contribute to the regulation of aerobic growth in *S. oneidensis* including CRP and ArcA. The 3',5'-cAMP phosphodiesterase (CpdA) appears to play a more significant role in aerobic growth than either CRP or ArcA, yet the deficiency does not appear to be the result of reduced oxidase genes expression. Interestingly, the ∆*cpdA* mutant was more deficient in aerobic respiration with several carbon sources tested compared to ∆*crp*, which was moderately deficient only in the presence of lactate. To identify the reason for ∆*cpdA* aerobic growth deficiency, we isolated a suppressor mutant with transposon insertion in SO_3550. Inactivation of this gene, which encodes an anti-sigma factor, restored aerobic growth in the *cpdA* mutant to wild-type levels. Inactivation of SO_3550 in wild-type cells, however, did not affect aerobic growth. The *S. oneidensis* genome encodes two additional CRP-like proteins that we designated CrpB and CrpC. Mutants that lack *crpB* and *crpC* were deficient in aerobic growth, but this deficiency was not due to the loss of oxidase gene expression.

## Introduction

*Shewanella oneidensis* MR-1 is a Gram-negative facultative anaerobe found mostly at the oxic/anoxic interface in freshwater environments. The ability of *S. oneidensis* to thrive in such environments is attributed to its extreme respiratory versatility with regard to electron acceptors, which include oxygen, radionuclides, elemental sulfur, and metal oxides (Fredrickson et al., 2008). The ability to use metals as terminal electron acceptors has spurred research into electricity generation by *S. oneidensis*. The location of *c*-type cytochromes, such as MtrC, on the outer cell surface (Beliaev et al., 2001) and the ability to reduce metal oxides made *S. oneidensis* and other metal reducing bacteria attractive candidates to study electricity production in microbial fuel cells (Bouhenni et al., 2010; Bretschger et al., 2010).

In addition to the anaerobic reductases, *S. oneidensis* expresses three terminal aerobic oxidases. These are encoded by the *cco* operon (SO_2364–2357), the *cox* genes (SO_4606–4609), and *cydABX* (SO_3286–3284). The *cco* and *cox* operons encode the *cbb_3_*- and *aa_3_*-type enzymes that belong to the heme-copper oxidase (HCO) family, while *cydAB* encodes a *bd*-type quinol oxidase (Borisov et al., 2011; Sousa et al., 2012; Le Laz et al., 2014).

Type A HCO or *aa*_3_-type cytochrome *c* oxidases have low oxygen affinity, and in many organisms, they are the primary enzymes used when oxygen is readily available (Ludwig, 1987; García-Horsman et al., 1994). In contrast, the *cbb*_3_-type cytochrome *c* oxidase has high affinity for oxygen, and it is the primary enzyme used under microaerobic conditions (Pitcher and Watmough, 2004; Buschmann et al., 2010). The *bd*-type enzymes are quinol oxidases that have a high affinity for oxygen, and as such, function under microaerobic conditions (Borisov et al., 2011).

The *S. oneidensis* aerobic oxidases do not appear to function under the same environmental conditions as their counterparts in other bacteria. The *cbb3*-type cytochrome oxidase, in addition to the *bd* quinol oxidase, appears to play a major role in aerobic respiration. Mutants deficient in *cbb3*-type cytochrome oxidase grew slower than the wild type, while loss of *bd* quinol oxidase did not have an effect on aerobic growth, suggesting that the *cbb3*-type enzyme is the major oxidase under aerobic conditions (Gao et al., 2010; Kouzuma et al., 2012; Le Laz et al., 2014). Mutants that lack *aa_3_*-type cytochrome *c* oxidase had no effect on aerobic growth and the lack of detectable enzyme activity in wild-type cells suggested that it may not be expressed (Le Laz et al., 2014). A recent report, however, showed that the *S. oneidensis aa_3_*-type cytochrome *c* oxidase is expressed under highly aerobic conditions and carbon starvation (Le Laz et al., 2016).

The cAMP-receptor protein CRP is a global regulatory protein that controls the expression of many of the anaerobic reductase genes (Pitcher and Watmough, 2004; Charania et al., 2009). ∆*crp* mutant is completely deficient in anaerobic growth with several electron acceptors and is slightly impaired in aerobic growth (Saffarini et al., 2003; Kasai et al., 2017). Three adenylate cyclases and one phosphodiesterase (CpdA) regulate cellular cAMP levels (Charania et al., 2009; Yin et al., 2016). Interestingly, mutants deficient in adenylate cyclase activity are deficient in anaerobic respiration, while a mutant that lacks CpdA is deficient in aerobic respiration (Charania et al., 2009; Yin et al., 2016). The work presented in this paper suggests that the *aa3*-type and *cbb3*-type cytochrome oxidases are involved in aerobic respiration when pyruvate and acetate are used as carbon sources. Furthermore, our results suggest that the aerobic growth deficiency observed in ∆*cpdA* is not due to the loss of cytochrome *c* oxidases, and this deficiency can be suppressed by a mutation in a putative anti-sigma factor.

## Materials and Methods

### Bacterial Strains and Growth Conditions

The bacterial strains and plasmids used in this study are listed in Table 1. *S. oneidensis* MR-1 and *Escherichia coli* cells were cultured aerobically in lysogeny broth (LB) at 30 and 37°C, respectively. Antibiotics (20mg/ml chloramphenicol, 25mg/ml kanamycin, and 25mg/ml gentamycin) were added as needed. For anaerobic growth, *S. oneidensis* cells were grown in basal medium supplemented with 50mM lactate, 0.02% casamino acids, and 10mM of either fumarate or DMSO (Saffarini et al., 2003). Anaerobic growth was performed in Hungate tubes and monitored at A_600_. To test aerobic growth, the mutants and the wild type were grown with vigorous shaking in 500ml flasks containing 50ml basal medium supplemented with 0.01% casamino acids, and 30 or 50mM of the indicated carbon source. For some experiments, growth was carried out in 24 well plates containing 600μl basal medium supplemented as above. Optical density was measured using the Tecan Infinite^®^ m200 PRO plate reader.

**Table 1:** List of strains and plasmids used in this study.

Strain/Plasmids	Description	References
*S. oneidensis*		
MR-1	Wild-type *S. oneidensis*	Fredrickson et al., 2008
SR-694	MR-1∆*crp*	Charania et al., 2009
SR-722	MR-1∆*cpdA*	This work
SR-1507	MR-1 ∆SO_2364–2,357 (∆*cco*)	This work
SR-1648	MR-1 ∆SO_4,606–4,609 (∆*cox*)	This work
SR-1649	MR-1 ∆*cco*∆*cox*	This work
SR-1699	MR-1 with pER21 insertion inSO_3,550	This work
SR-1700	∆*cpdA* with pER21 insertion inSO_3,550	This work
SR-1716	MR-1 ∆*cydAB* (∆*cyd*)	This work
SR-1717	MR-1 ∆SO-2551	This work
SR-1718	MR-1 ∆SO_2550–2,551	This work
*E. coli*		
EC100D+	*E. coli* EC100 derivative, *pir*+	Epicenter Technologies
β2155	*pir*::RP4, Km^r^	Dehio and Meyer, 1997
**Plasmids**		
pJBC1	Cloning and sequencing vector, Cm^R^	Bouhenni et al., 2010
pER21	R6K *ori*, Gm^R^, *sacB*, *lacZ* a-fragment	Bouhenni et al., 2005
pMC10	Promoter probe vector, *lacZ*, Cm^R^	Shroff et al., 2010
pAB1	*cpdA* in pJB3Cm6	This work
RB1	mini*Himar* Transposon	Bouhenni et al., 2005

### Generation of Chromosomal Deletion Mutants

Chromosomal gene deletions were performed as described previously (Shirodkar et al., 2011). 1kb fragments flanking the gene(s) of interest were amplified using Phusion polymerase (New England Biolabs) and the primers listed in Supplementary Table S1. The fragments were amplified, digested, and then ligated before cloning into the SmaI site of the suicide vector pER21 (Table 1). Plasmids carrying the insert of interest were used to transform *E. coli* β2155 cells (Dehio and Meyer, 1997) by electroporation and then transferred to *S. oneidensis* MR-1 strains by conjugation. Mutants with chromosomal deletions were selected on 5% sucrose plates then screened by PCR to verify deletion of the target gene.

### Promoter-Activity Assays

DNA fragments directly upstream of the genes of interest were amplified from genomic DNA by PCR using Phusion polymerase and the primers listed in Supplementary Table S1. HindIII and BamHI restriction sites were included in the primer design. The amplified fragments were digested and cloned into pMC10 (Shroff et al., 2010). Following transformation of *E. coli* β2155 (Dehio and Meyer, 1997), the plasmids were transferred into wild-type and mutant strains by conjugation. Cultures were assayed for β-galactosidase activity essentially as previously described (Miller, 1972).

### cAMP Detection Assay

*Shewanella oneidensis* strains grown in LB overnight were diluted 1:8 with basal medium supplemented with 50mM lactate and 0.02% casamino acids and used to grow cultures aerobically and anaerobically. For anaerobic growth, cultures were supplemented with 10mM fumarate or 10mM DMSO and incubated anaerobically in sealed serum vials for 3h. For aerobic growth, 50ml cultures in 2liter flasks were grown for 3h with vigorous shaking. Cells were then pelleted and lysed by boiling in phosphate buffered saline. The supernatants from the boiled samples were used to measure intracellular cAMP using the Fluorescent HitHunter cAMP detection kit (GE-Healthcare). Protein concentrations in equivalent culture samples were determined using the BCA assay reagents (Thermo Scientific). Spent media from both aerobic and anaerobic cultures were filtered with a 0.22μm filter then used to measure extracellular cAMP levels.

### Generation of ∆*cpdA* Suppressor Mutant

Transposon mutants in a ∆*cpdA* background were generated using pmini*Himar* RB1 (Bouhenni et al., 2005). The plasmid was transferred into *S. oneidensis* by conjugation. Mutants were selected on basal medium agar supplemented with 30mM lactate, 0.01% casamino acids, and 25μg/ml kanamycin. Chromosomal DNA was isolated, digested with BamHI, re-ligated, and used to transform *E. coli* EC100D^+^. Plasmid DNA was sequenced by Eurofins Scientific using the primer 615 (Supplementary Table S1). The resulting sequences were analyzed, and mutants of interest were selected for further study. Insertional inactivation of the gene of interest was achieved by cloning an internal fragment of 500bp into the suicide plasmid pER21 (Bouhenni et al., 2010). The resulting plasmid was transferred into wild-type MR-1 and ∆*cpdA* by conjugation. Mutants were selected on LB agar plates supplemented with 25mg/ml gentamycin.

## Results

### The Effect of Carbon Source on Growth of *S. oneidensis* Oxidase Mutants

**Figure 1:**
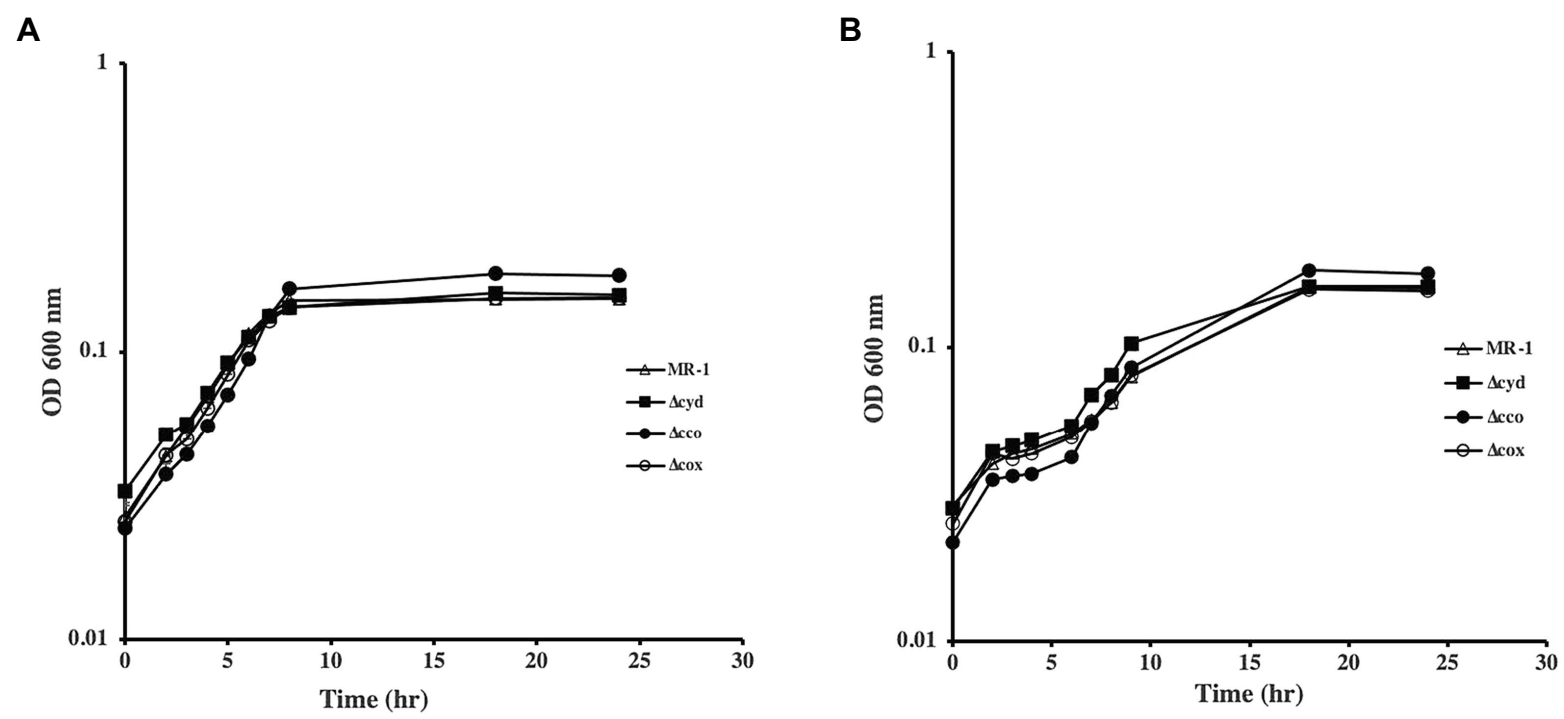
Anaerobic growth of oxidase mutants in lactate-minimal medium with fumarate **(A)** and DMSO **(B)**. None of the mutants exhibited growth deficiencies under the conditions tested. The data are the means and standard deviations of three independent experiments.

**Figure 2:**
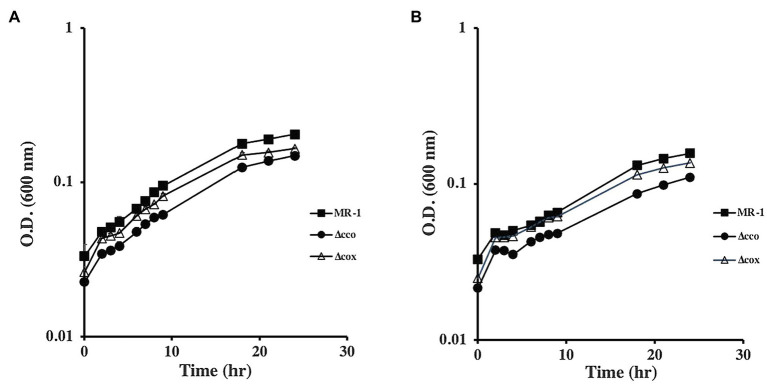
Anaerobic growth of oxidase mutants with fumarate **(A)** and DMSO **(B)** as electron acceptors and pyruvate as the carbon source. ∆*cox* grew similar to the wild type, while ∆*cco* had a moderate growth deficiency with both electron acceptors. ∆*cyd* mutant grew similar to the wild type but is not shown for clarity. The data are the means and standard deviations of three independent experiments.

To understand the role of the *S. oneidensis* oxidases, we generated mutants that lack the *cco*, *cox*, and *cyd* operons that encode the *cbb_3_*-type oxidase, *aa_3_*-type oxidase, and *bd* quinol oxidase, respectively. The mutants were tested for growth using lactate, pyruvate, or acetate as carbon and energy sources. In the absence of oxygen, with either fumarate or DMSO as electron acceptors, all mutants grew similar to the wild type when lactate was used as the carbon source (Figure 1). The ∆*cco* mutant was slightly deficient in anaerobic growth with DMSO and fumarate when pyruvate was the sole carbon source and electron donor. Growth of the other oxidase mutants tested was indistinguishable from that of the wild type (Figure 2 and data not shown).

**Figure 3:**
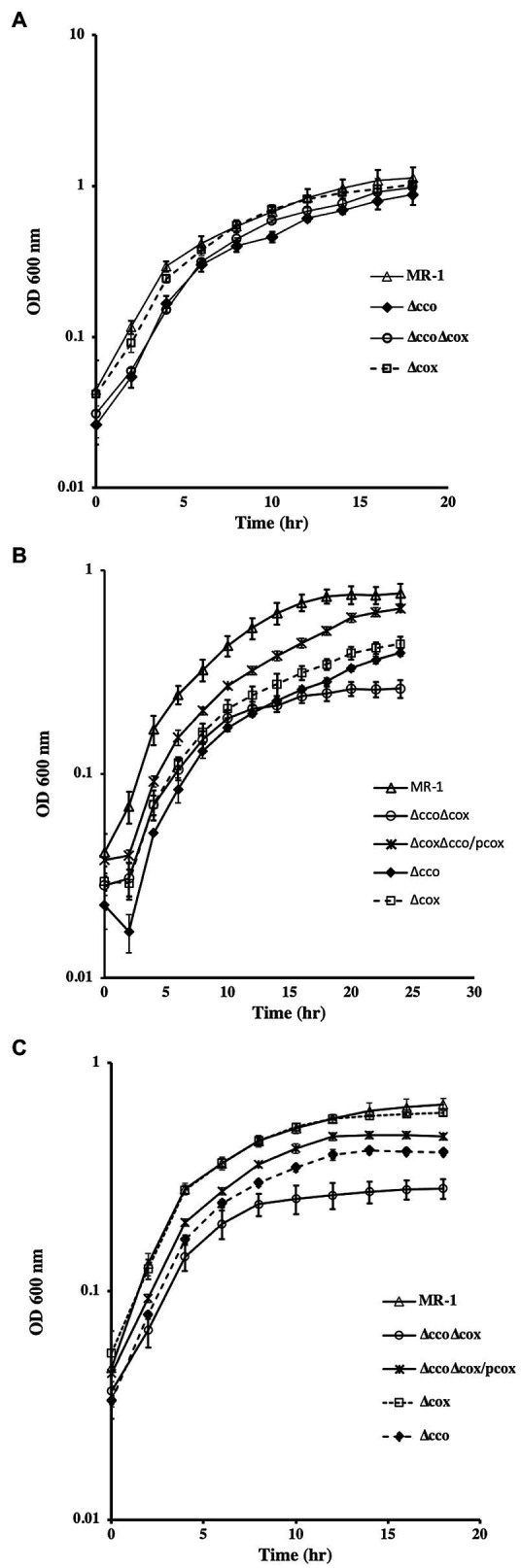
Aerobic growth of *S. oneidensis* wild-type and oxidase mutants with lactate **(A)**, acetate **(B)**, and pyruvate **(C)**. Both ∆*cco* and ∆*cco*∆*cox* mutants were more deficient in growth with acetate and pyruvate than with lactate. ∆*cyd* mutant grew similar to the wild type but is not shown for clarity. The data are the means and standard deviations of three independent experiments.

Single and double oxidase mutants were tested for aerobic growth using different carbon sources. In the presence of lactate, deletion of *cco* genes slowed growth of the mutant, whereas deletion of the other oxidase genes had no effect (Figure 3A). The estimated doubling time of ∆*cco* mutant was 6.7h. The doubling time of ∆*cyd*, ∆*cox*, and wild-type strains ranged between 5.0 and 5.4h. In contrast, significant growth deficiencies were observed in *cco* and *cox* single and double mutants when grown with acetate or pyruvate (Figures 3B,C). Overall, growth is slower when acetate or pyruvate are used as sole carbon sources, likely exacerbating the deficiency phenotype. In the presence of acetate, the doubling time of ∆*cco*, ∆*cox*, and ∆*cco*∆*cox* mutants was 1.35, 1.3, and 1.5 times that of the wild type, respectively. A similar growth pattern was seen when the mutants were grown aerobically with pyruvate. It is noteworthy that the deficiency of the double ∆*cco*∆*cox* was more pronounced with a doubling time almost twice that of the wild type. Introduction of *cox* genes into the double mutant restored its ability to grow similar to the single *∆cco* mutant (Figure 3C). These results suggest that the *aa_3_*-type oxidase is functional in *S. oneidensis*, but its activity and contribution to aerobic growth may depend on the energy and carbon source that the bacterium uses.

### The Role of CRP and CpdA in Aerobic Growth

**Figure 4:**
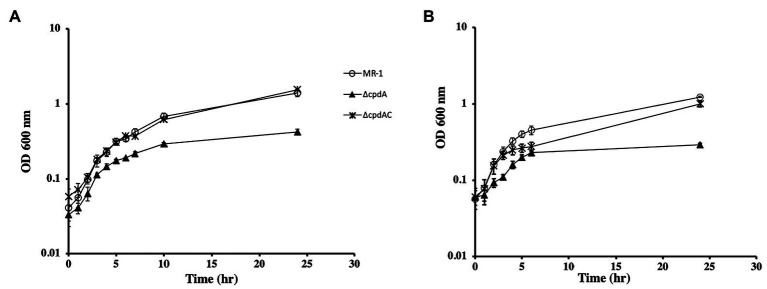
Aerobic growth of wild type, ∆*cpdA*, and complemented mutant (∆*cpdA*C) in basal medium supplemented with lactate **(A)** or pyruvate **(B)**. Complementation of ∆*cpdA* restored its ability to grow with both carbon sources. Legend applies to **A** and **B**. The data are the means and standard deviations of three independent experiments.

The function of CpdA (SO_3901) as a cAMP phosphodiesterase has been previously described (Yin et al., 2016; Kasai et al., 2018). We generated a ∆*cpdA* mutant and tested for cAMP levels and growth phenotypes. In aerobically grown cells, intracellular cAMP increased from 28.9±3.1 pmoles/mg protein in the wild type to 106.2±20.3 pmoles/mg protein in ∆*cpdA*. Similarly, higher extracellular cAMP concentrations were consistently detected in the ∆*cpdA* mutant than in the wild type (26.5±3.3 and 13.8±2.5 nmoles/mg protein, respectively). These results are in agreement with the previously reported role of CpdA as a cAMP phosphodiesterase (Yin et al., 2016; Kasai et al., 2018). Because cAMP is required for CRP activation, we reasoned that a *cpdA* mutation will affect anaerobic respiration. Growth of ∆*cpdA* in lactate-basal medium with fumarate or DMSO was similar to the wild type but appeared to reach stationary phase at a lower cell density than the wild type (Supplementary Figure S1 and data not shown). Growth of ∆*cpdA* was also tested aerobically in rich and minimal media. We did not detect a growth deficiency when the mutant was grown aerobically in LB (data not shown). However, the mutant grew poorly in minimal medium supplemented with lactate (Figure 4). These results are in agreement with the findings of Kasai et al. (2018). We further tested aerobic growth of ∆*cpdA* and complemented mutant in the presence of pyruvate and acetate (Figure 4 and Supplementary Figure S2). The mutant was deficient in aerobic growth in the presence of these carbon sources and complementation restored its ability to grow similar to the wild type (Figure 4 and data not shown).

Although *S. oneidensis* CRP is best known for its role in the regulation of anaerobic respiration, it appears to have an effect on aerobic respiration. To further investigate the role of CRP in this process, we monitored aerobic growth of wild type and ∆*crp* mutant in the presence of different carbon sources. In the presence of lactate, the ∆*crp* mutant exhibited a slight deficiency in growth compared to the wild type (Supplementary Figure S2). This deficiency was not observed when acetate or pyruvate was used as carbon sources or when the mutant was grown in rich medium, such as LB (Supplementary Figure S2 and data not shown). This result was surprising, and it is not clear why the *crp* mutant exhibits growth deficiencies with lactate but not with other carbon sources.

**Figure 5:**
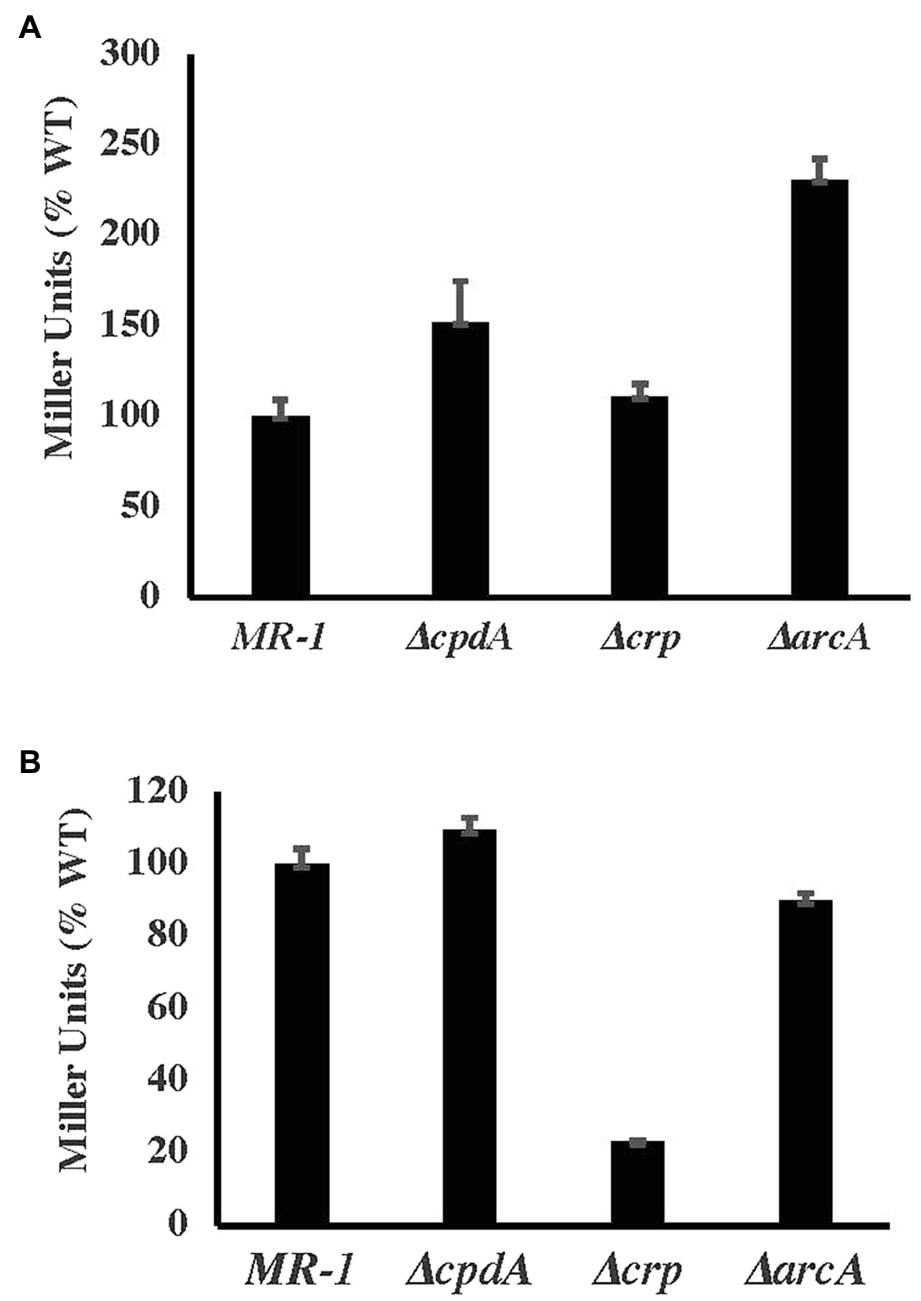
ß-galactosidase activity (in Miller relative to WT) of *cco* **(A)** and *cyd* **(B)** promoter-*lacZ* fusions under aerobic conditions in basal medium supplemented with 30mM lactate and 0.01% casamino acids.

Previous reports suggested the positive regulation of *cco* and *cyd* genes by CRP and CpdA (Yin et al., 2016). A more recent microarray analysis suggested that CpdA does not an effect expression of these two oxidases (Kasai et al., 2018). Using *lacZ*-promoter fusions, we tested the expression of *cco* and *cyd* genes in ∆*crp* and ∆*cpdA*. Our results indicate that CpdA and CRP do not affect the expression of the *cco* operon (Figure 5). Furthermore, loss of CRP, but not CpdA, led to reduced expression of *cydAB* indicating that this operon is positively regulated by CRP (Figure 5).

### Isolation of a ∆*cpdA* Suppressor Mutant

**Figure 6:**
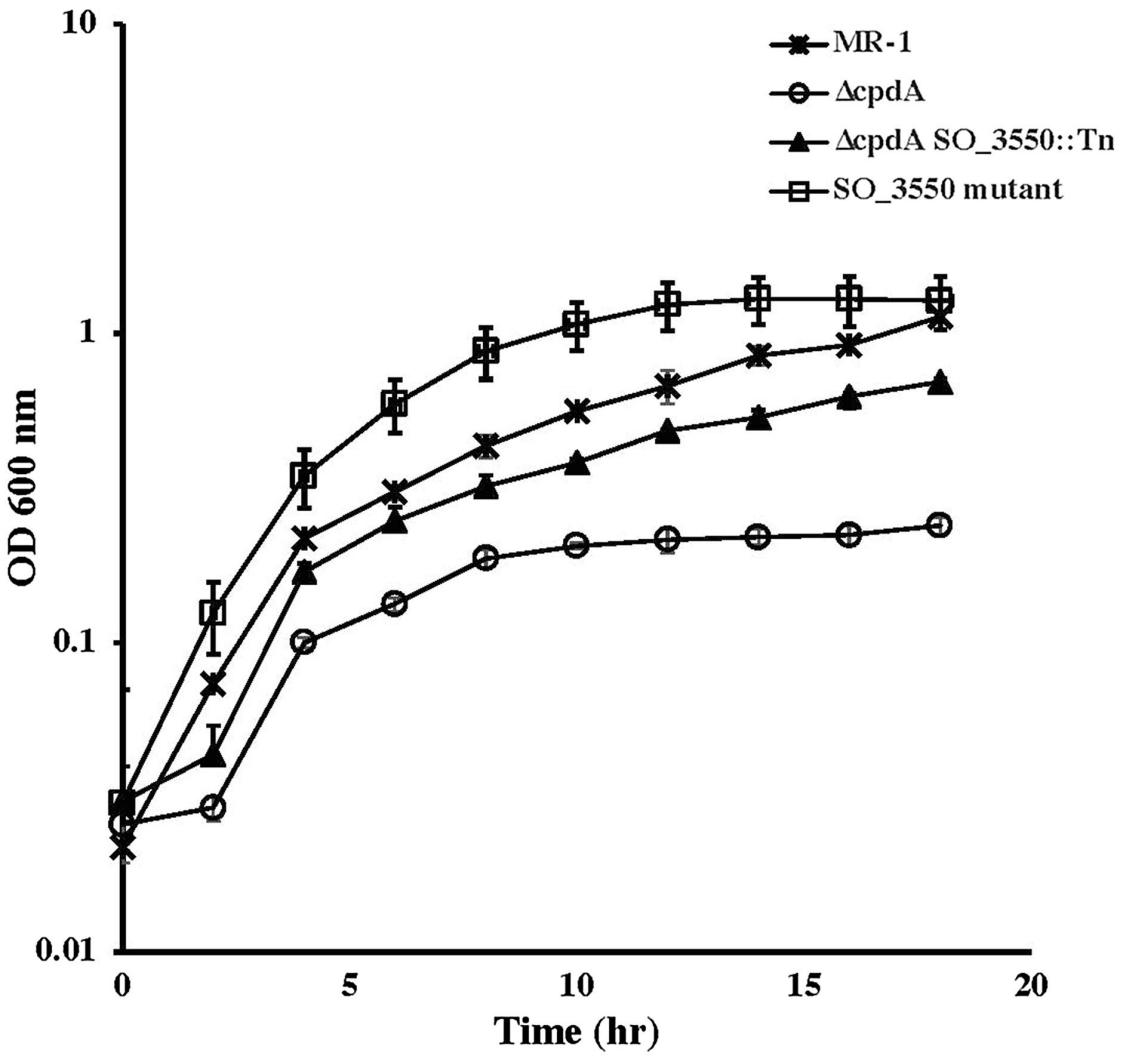
Aerobic growth of wild-type *Shewanella oneidensis* and ∆*cpdA* strains in basal medium supplemented with 30mM lactate and 0.01% casamino acids. Insertional inactivation of SO_3550 in ∆*cpdA* allowed the phosphodiesterase mutant to recover its ability to grow aerobically in minimal media. Inactivation of SO_3550 in wild-type MR-1 did not affect the ability of the mutant to grow aerobically. The data are the means and standard deviations of three independent experiments.

To identify the reason for ∆*cpdA* growth deficiency under aerobic but not anaerobic conditions, we isolated a transposon mutant in a ∆*cpdA* background that grew in basal medium under aerobic conditions similar to the wild type (Figure 6). Sequence analysis identified a transposon insertion in SO_3550, which is predicted to encode an anti-sigma factor and is in an operon with SO_3551 that encodes a putative extracytoplasmic function (ECF) sigma factor. To confirm that the ability of ∆*cpdA* SO-3550::*himar* to grow in minimal medium aerobically was due to inactivation of SO_3550, we generated an insertional mutation in this gene in a ∆*cpdA* background. Inactivation of SO_3550 in ∆*cpdA* restored the mutant’s ability to grow aerobically in minimal medium (Figure 6). Insertional inactivation of SO_3550 was also performed in wild-type MR-1 in an attempt to identify SO_3550 function. However, analysis of the resulting mutant did not reveal any growth deficiencies under the conditions we tested (Figure 6).

### Identification of Putative cAMP-Binding Proteins Involved in Aerobic Respiration

**Figure 7:**
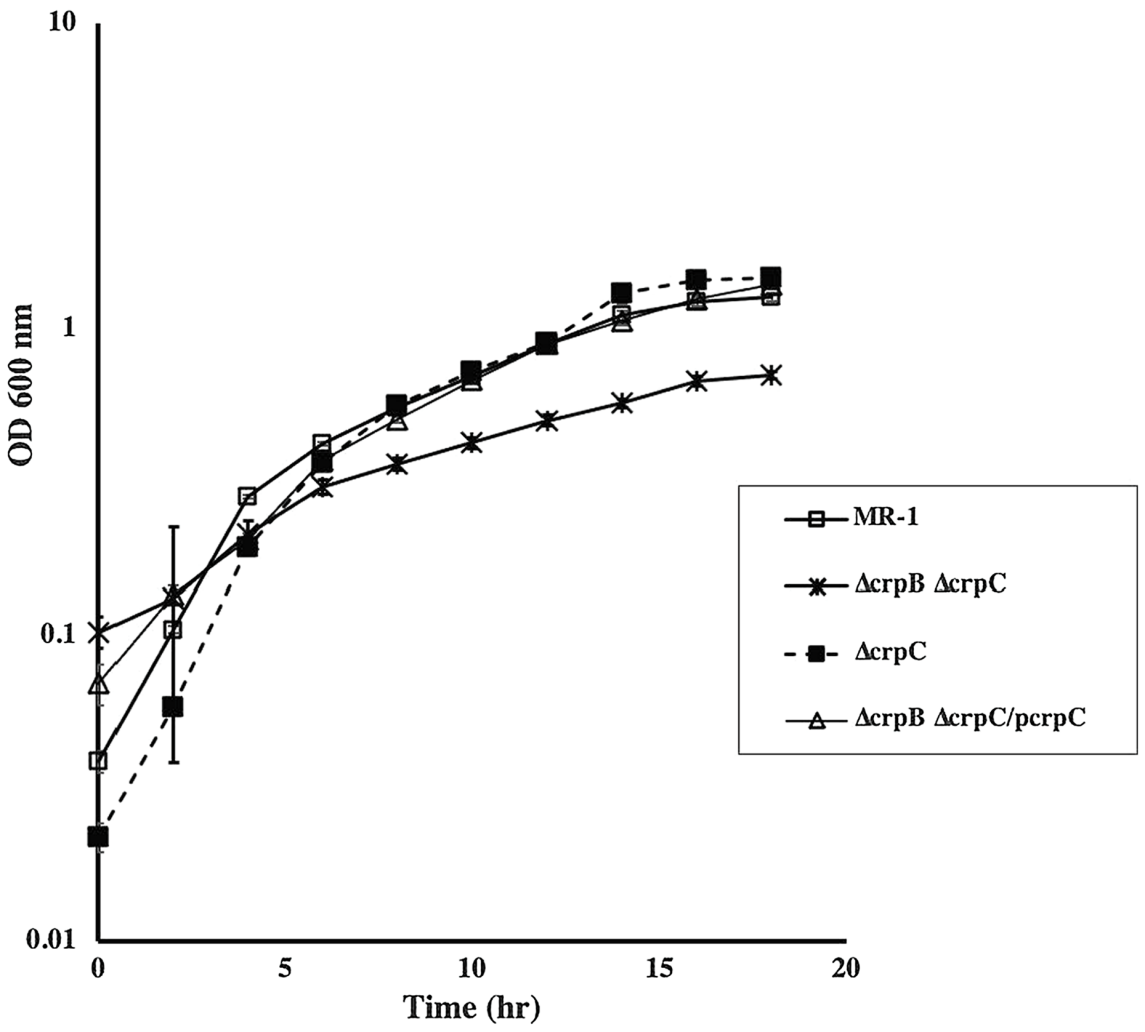
Aerobic growth of wild type, ∆*crpB* and ∆*crpC* with lactate as the carbon source. Loss of both genes resulted in slow growth compared to the wild type. Growth was restored to wild-type levels by the introduction of *crpC*. Complementation was also achieved by the introduction of *crpB* and *crpC* on a plasmid (data not shown). The data are the means and standard deviations of three independent experiments.

In addition to CRP, the *S. oneidensis* genome contains two genes, SO_2550 and SO_2551, that we designated *crpB* and *crpC, respectively*. These genes are separated by 125 nucleotides. Sequence analysis identified putative nucleotide and DNA-binding domains with similarity to CRP-like proteins. Single and double chromosomal deletion mutants were generated and tested for aerobic growth with different electron acceptors. ∆*crpC* grew aerobically with all carbon sources tested. Similarly, a mutant that lacks *cprB* did not exhibit any aerobic growth deficiency. In contrast, a strain that lacked both genes was deficient in aerobic growth with lactate, pyruvate, and acetate. These results suggest that *crpB* and *crpC* may be redundant and both play a role in aerobic growth. Complementation of the mutants restored their ability to grow with all carbon sources tested (Figure 7). Since the double ∆*crpB ∆crpC* mutant was deficient in aerobic growth, we tested it for expression of *cco* and *cyd* genes using promoter-*lacZ* fusions. There was no detectable difference in ß-galactosidase activity in the mutant compared to the wild type, suggesting that neither CrpB nor CrpC regulate the expression of the *cco* and *cyd* genes (Supplementary Figure S3).

## Discussion

*Shewanella oneidensis* exhibits amazing respiratory versatility that allows it to survive in the changing oxic/anoxic interface of aquatic environments. At least 14 terminal electron acceptors have been identified and many anaerobic terminal reductases and reduction pathways have been elucidated (Beliaev et al., 2002; Fredrickson et al., 2008; Gao et al., 2009; Shirodkar et al., 2011; Brockman et al., 2020). For aerobic respiration, three terminal oxidases have been identified. The *cbb_3_*-type is active under oxygen replete conditions and appears to be the major oxidase (Le Laz et al., 2014) while the *bd*-type quinol oxidase, encoded by *cydAB*, functions under microaerobic conditions and early stationary phase (Zhou et al., 2013; Le Laz et al., 2014). The function of the *aa3*-type was more difficult to identify. Earlier reports suggested this cytochrome *c* oxidase does not play a role in aerobic respiration and may not be expressed (Zhou et al., 2013). Biochemical analysis failed to detect *aa3*-type oxidase in aerobic cells grown in rich media (Le Laz et al., 2014), but the enzyme was detectable in cells grown in minimal medium but starved for nutrients similar to the *Pseudomonas aeruginosa aa_3_*-type oxidase (Kawakami et al., 2010; Le Laz et al., 2016; Osamura et al., 2017). Kouzuma et al. (2012) isolated a mutant deficient in the *aa_3_*- and *cbb_3_*-type oxidases that grew slower than the wild type or the single mutants on lactate (Kouzuma et al., 2012) suggesting that the *aa_3_*-type cytochrome *c* oxidase activity is not confined to conditions of extreme nutrient stress. Our results support this prediction. In defined minimal media supplemented with lactate, little difference was observed between the wild-type and the single oxidase mutants (Figure 3). However, the growth patterns of these mutants appear to change dramatically in the presence of other carbon courses. The ∆*cco* mutant was clearly deficient in aerobic growth with pyruvate and acetate. The ∆*cox* mutant had a significant deficiency when grown with acetate, and the double ∆*cco*∆cox was noticeably deficient in growth in the presence of both carbon sources (Figure 3). Lactate is the preferred carbon source for *S. oneidensis* and in its presence, acetate metabolism is inhibited (Tang et al., 2007). Based on the results presented in this paper and work from others, we propose that the *S. oneidensis aa_3_*-type cytochrome oxidase is active under nutrient stress in the absence of its preferred carbon source. It may be that expression of *aa_3_*-type cytochrome oxidase constitutes part of a stress response to nutrient or oxidative stress conditions. We also observed that the ∆*cco* strain was mildly deficient in anaerobic growth with fumarate & DMSO as electron acceptors (Figure 2). This result backs the findings of Gao et al. (2010) who observed that the ∆*cco* mutant was deficient in Uranium reduction.

Carbon metabolism in *E. coli* and other bacteria is regulated by the cAMP-receptor protein CRP (Guest, 1992). In *S. oneidensis* and its close relative *Shewanella* ANA-3, CRP regulates anaerobic respiration (Saffarini et al., 2003). *S. oneidensis* ∆*crp* mutants were deficient in aerobic growth with lactate but were not significantly deficient with acetate or pyruvate (Kasai et al., 2017 and Supplementary Figure S2). The aerobic growth deficiency in ∆*crp* does not appear to be due to loss of oxidase genes since expression of the *cco* operon is not affected by loss of CRP (Figure 5). *cydAB* expression was downregulated in ∆*crp*, but because loss of *cydAB* does not significantly affect aerobic growth, we do not expect this regulation to be the reason for the *crp* mutant deficiency. In addition to CRP, ArcA, which regulates anaerobic DMSO reduction in *S. oneidensis*, appears to be involved in aerobic growth. *arcA* mutants grow slower than the wild type under aerobic conditions (Gralnick et al., 2005; Shroff et al., 2010). These results are surprising because ArcA appears to negatively regulate *cco* gene expression under both aerobic and anaerobic conditions (Figure 5 and Shroff et al., 2010).

cAMP phosphodiesterases regulate gene expression by controlling intracellular cAMP levels. These enzymes are involved in colony size, virulence, regulation of carbon metabolism, and biofilm formation among others (for review see Matange, 2015). Their function can be dependent or independent from the cAMP-receptor protein and may include hydrolysis of other cyclic nuclides, such as cGMP (Matange, 2015). *S. oneidensis* CpdA hydrolyzes cAMP and cyclic di-GMP (This work; Yin et al., 2016; Kasai et al., 2018). Although *S. oneidensis* CpdA is involved in the regulation of cAMP levels under both aerobic and anaerobic conditions, it does not appear to play a significant role in anaerobic growth, unlike CRP (Supplementary Figure S1). In contrast, *S. oneidensis cpdA* mutants are deficient in aerobic growth. This deficiency was observed in minimal (Figure 4) but not in rich media (Kasai et al., 2018). Although previous reports suggested that CpdA positively regulates the expression of aerobic oxidase genes, our results (Figure 5) and those of Kasai et al. (2018) indicated that expression of the oxidase genes was not affected in ∆*cpdA*. In fact, the aerobic growth deficiency in *cpdA* mutant can be complemented by the addition of casamino acids (Supplementary Figure S4 and Kasai et al., 2018). It is interesting to note that growth of ∆*cpdA* with 0.2% casamino acids was similar to growth of the wild type with 0.05% casamino acids. This suggests that the mutant, although it can be complemented with casamino acids, is still deficient compared to the wild type (Supplementary Figure S4).

To further understand the role of CpdA in aerobic respiration, we isolated a suppressor mutant of *cpdA* with an insertion in SO_3550 that is predicted to encode an anti-sigma factor. This gene is located downstream of SO_3551, which is predicted to encode an ECF sigma factor. The two genes appear to be arranged in an operon with another ORF (SO_3552). Since SO_3550 is the last gene in the presumptive operon, its disruption is not expected to affect the expression of the two other ORFs, suggesting that loss of only the anti-sigma factor is sufficient to restore aerobic growth to the *cpdA* mutant to almost wild-type levels (Figure 6). The function of SO_3550 is not clear. We attempted to identify its function by inactivating this gene in the wild-type strain, but we did not detect a deficiency in growth when compared to the wild type. It is intriguing that SO_3550 is adjacent to an ECF sigma factor gene and that sequence analysis predicts possible regulation of this operon by CRP. The microarray data presented by Kasai et al. (2018) do not indicate up- or downregulation of any of the five MR-1 ECF sigma factors or their cognate anti-sigma factors in the ∆*cpd*A mutant (Kasai et al., 2018). Therefore, the action of this anti-sigma factor and its cognate sigma may not be directly affected by *cpd*A. ECF sigma factors are typically activated in response to stress. In view of the fact that ∆*cpd*A growth deficiency can be ameliorated by either the addition of amino acids (Supplementary Figure S4 and Kasai et al., 2018) or inactivation of SO_3550, we suggest that the growth deficiency may be due to energy stress and lack of sufficient ATP. This may be reflected in the downregulation of amino acid biosynthesis genes in ∆*cpdA* (Kasai et al., 2018). In the absence of a cAMP phosphodiesterase, the cell accumulates cAMP and may deplete ATP normally used for biosynthesis and growth. Inactivation of SO_3550 is predicted to activate the ECF sigma factor SO_3551. SO_3551 may modulate the function of a subset of adenylate cyclases under stress conditions or play a role in the regulating of amino acid metabolism. Clearly, additional work is needed to elucidate the function of SO_3550 and its putative cognate sigma factor SO_3551.

Many *S. oneidensis* genes exhibit redundancy. Genes involved in metal reduction, for example, have multiple homologs in the genome. There are three adenylate cyclases whose functions appear to be partially redundant. There is also redundancy in the function of terminal oxidases, such as the *cbb_3_*- and *bd*- type oxidases. CRP is no exception. The *S. oneidensis* genome encodes two putative cAMP-receptor-like proteins. Similar to CRP and ArcA, CrpB and CrpC play a role in aerobic growth (Figure 7) but do not appear to have an effect on the expression of terminal aerobic oxidase genes (Supplementary Figure S3). We suggest that these CRP-like proteins have a role in carbon metabolism similar to CRP (Kasai et al., 2017). The results presented here add to our knowledge of the mechanisms of aerobic respiration in *S. oneidensis*, but also add new elements to the complexity of the process. Clearly additional work is needed to understand how *S. oneidensis* regulates growth and respiration in its environment.

## Data Availability Statement

The original contributions presented in the study are included in the article/Supplementary Material, and further inquiries can be directed to the corresponding author.

## Author Contributions

KB generated oxidase mutants and analyzed the mutant’s phenotypes and gene expression. AB generated cpdA mutant, measured cAMP level in aerobic and anaerobic cells, and analyzed the mutant’s phenotypes. DS conceived the project, performed growth experiments, and wrote the manuscript. All authors contributed to the article and approved the submitted version.

## Conflict of Interest

The authors declare that the research was conducted in the absence of any commercial or financial relationships that could be construed as a potential conflict of interest.

## Publisher’s Note

All claims expressed in this article are solely those of the authors and do not necessarily represent those of their affiliated organizations, or those of the publisher, the editors and the reviewers. Any product that may be evaluated in this article, or claim that may be made by its manufacturer, is not guaranteed or endorsed by the publisher.
