# Supplementary material for: Aerobic Respiration and Its Regulation in the Metal Reducer Shewanella oneidensis
Source: Front Microbiol. 2021 Sep 9;12:723835. doi: 10.3389/fmicb.2021.723835 (PMC8458880; doi:10.3389/fmicb.2021.723835)
Supplement: Supplementary file 1 [file Data_Sheet_1.PDF]

Table S1. List of primers used in this study

Primer	Sequence
**Primers for *cpdA* deletion**	
**Upstream primers**	
3901F1	5'-CACGTTCAAGTCAACTGCG-3'
3901R1	5'-GTA**GGATCC**TCCCTCATCACTATGATACATACG-3'
**Downstream primers**	
3901F2	5'-GTA**GGATCC**GACCGCAGCTTGAGGAAC-3'
3901R2	5'-GCACTGTGACTTCGACTC-3'
**Primers for SO_3550 insertion**	
3550insF	5'-GCATCATCCGCAGCAACTTTCG-3'
3550insR	5'-GGCGTTACACCAAGAAGTCTCAG-3'
**Primers for SO_2550-1 deletion**	
2550F	5'-CATCATAGTTCAGCTAAAACAGCG-3'
2550R	5'-GATCGGATCCGTGTGAATTAGCTGCTTATTCG-3'
2551R1	5'-GATCGGATCCGCAAATACCATAGCAACTTCAGTC-3'
2551R	5'-GATCGGATCCGGTTAACAACTACAGC-3'
**Primers for SO_2551 deletion**	
2551F	5'-GCTTAAGCGTAGCGAGATTAATGTTG-3'
2551F1	5'-GATCGGATCCGCAAATACCATAGCAACTTCAGTC-3'
2551R	5'-GATCGGATCCGGTTAACAACTACAGC-3'
2551R1	5'-CCATTGCTTTAGTTGCCGTAGC-3'
**Primers for SO_2550-1 complementation**	
2550fNDE	5'-GATCCATATGTCTCAAAACGCCCTAG-3'
2551rBAM	5'-GATCGGATCCGGCAGGTTTATACAGG-3'
**Primers for SO_2551 complementation**	
2551fNDE	5'-GATCCATATGGATTGAAAGTAGTGTC-3'
2551rBAM	5'-GATCGGATCCGGCAGGTTTATACAGG-3'
**Primers for *cco* deletion**	
2357F	5'-GATCGGATCCCCCAAGCGAATGAATT-3'
2357R	5'-CCACACGCTGATCAATCAC-3'
2364F	5'-GCAGATGTGTTCGATGCCCTTTC-3'
2364R	5'-GATCGGATCCGCTTCCACTTATT-3'
**Primers for *cyd* deletion**	
CydABF1	5'-CATGCTCAAGAGGGTGCC-3'
CydABF2	5'-GATCGGTACCGGTTCCAATTGCTACG-3'

CydABR1	5’-GATCGGTACCCCAAATCGCTAGATCC-3’
CydABR2	5’-CTCGATTTGAAATATGCCG-3’

Primers for *cox* deletion

SO4606-9F2	5’-ATCGAAGCTTCTCGACCTAAGTAACG-3’
SO4606-9R1	5’-ATCGAAGCTTCACCTCAACTCTAGC-3’
SO4606-9R2	5’-CCGCCGTGATACCAATGC-3’
SO4606F1	5’-CCGAAAATGAAGACTACTCACC-3’

Primer for sequencing

Primer 615	5’-TCGGGTATCGCTCTTGAAGGG-3’

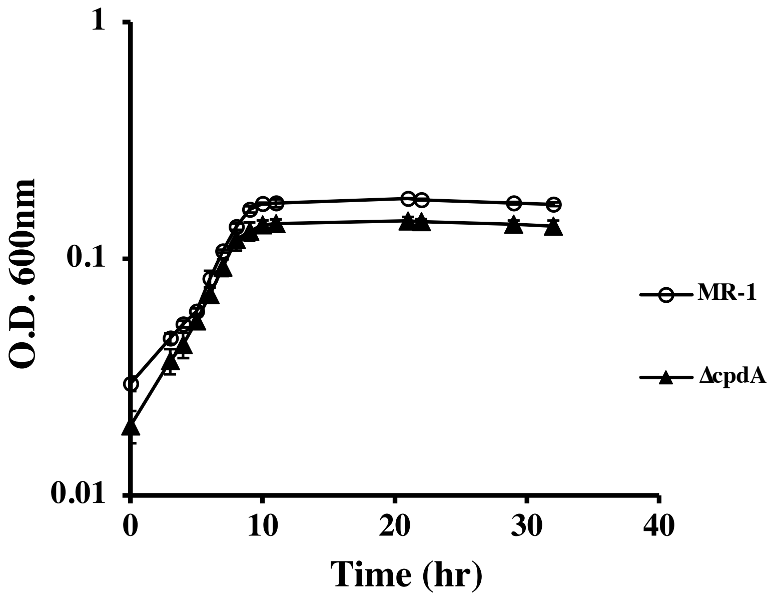

Figure S1. Anaerobic growth of wild type and *cpdA* mutant with lactate as the carbon source and DMSO as the electron acceptor. The mutant grew similar to the wild type but reached a lower density at stationary phase. Error bars represent standard deviation.

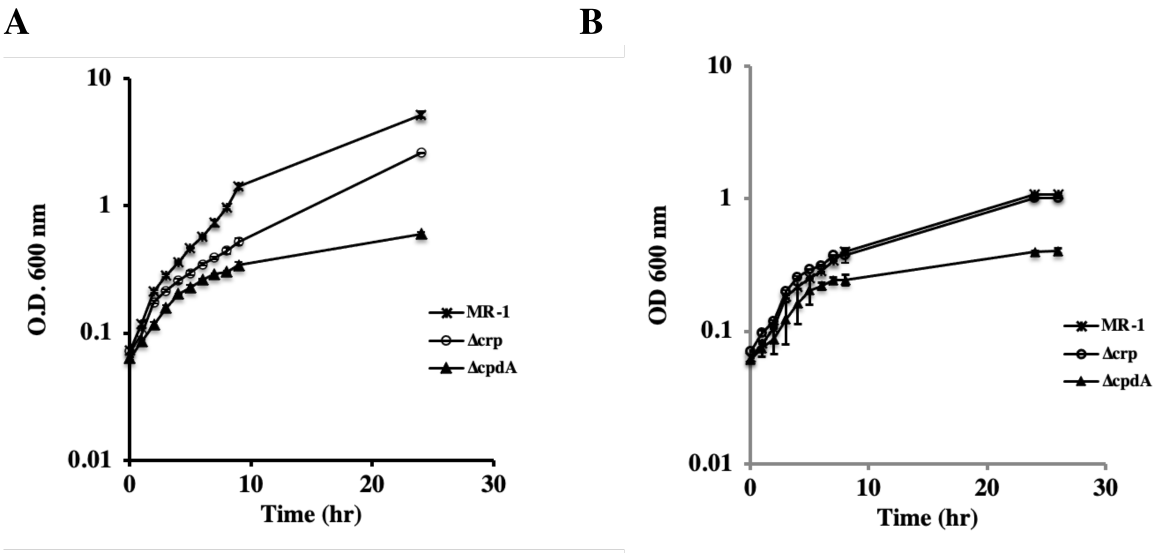

Figure S2. Aerobic growth of wild type, Δ*crp* and Δ*cpdA* in minimal medium supplemented with lactate (A) or acetate (B). Δ*crp* was deficient in growth with lactate but not acetate in contrast to Δ*cpdA* that was deficient in both. Growth with pyruvate gave similar results to growth with acetate (data not shown). Error bars represent standard deviation.

A

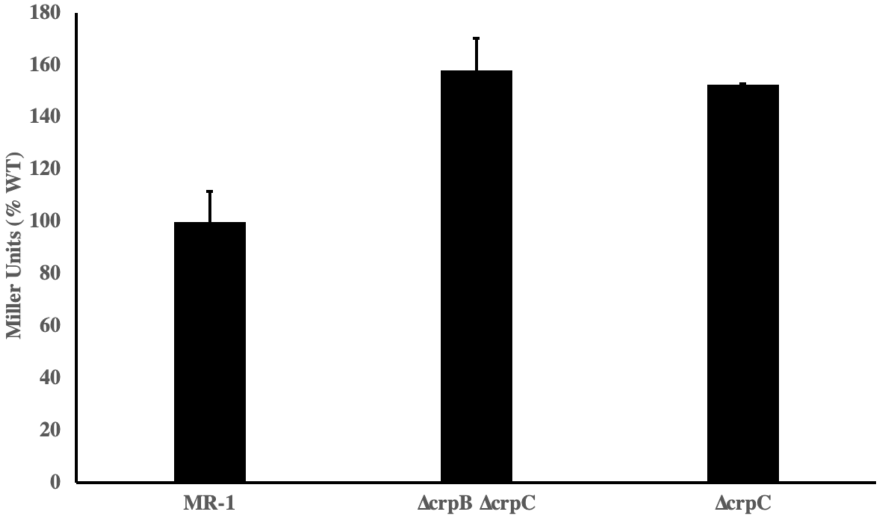

B

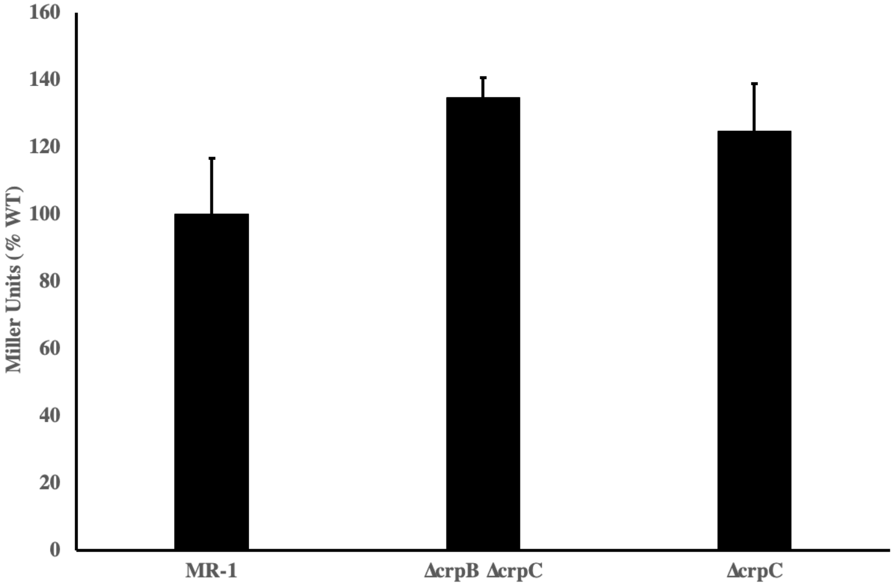

Figure S3. β-galactosidase activity (in Miller relative to WT) of *cco* (A) and *cyd* (B) promoter-*lacZ* fusions under aerobic conditions in wild type, Δ*crpB*Δ*crpC*, and Δ*crpC*. Expression of both promoters was not significantly affected by the deletion of the putative *crp* genes. Error bars represent standard deviation.

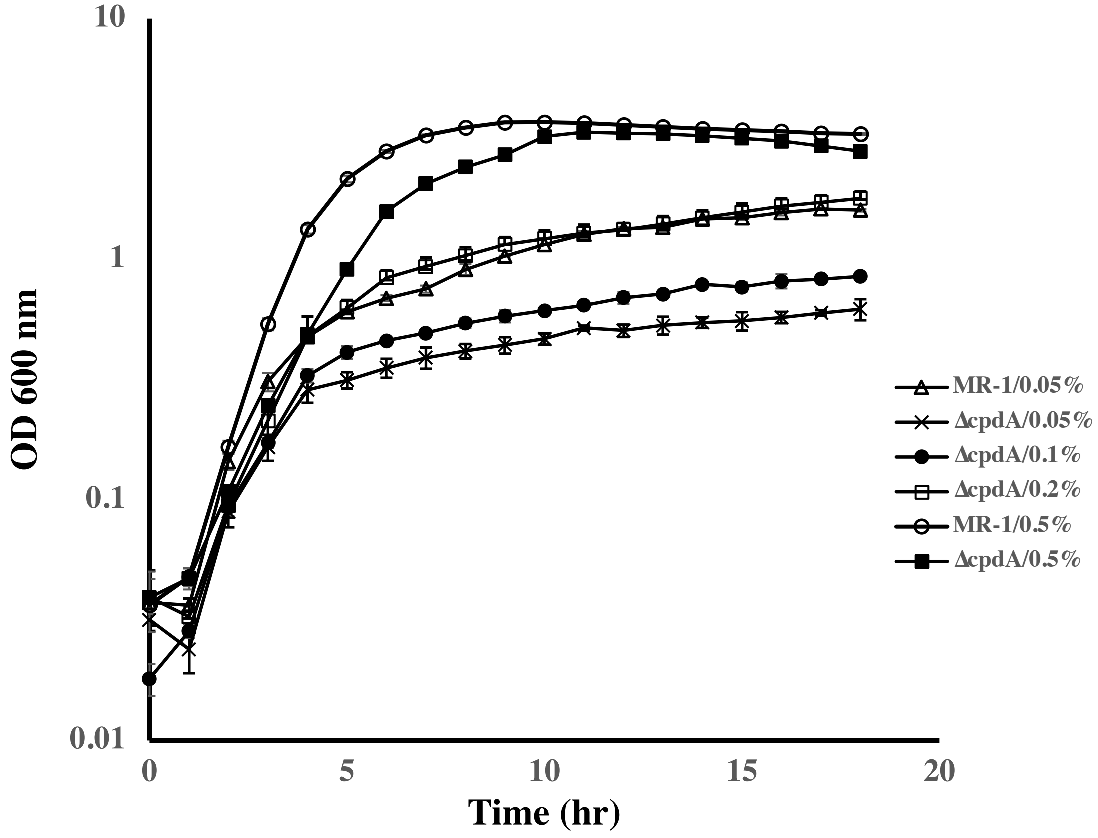

Figure S4. Growth of wild type and Δ*cpdA* mutant in minimal medium supplemented with lactate and the indicated amounts of casamino acids. Note that growth of Δ*cpdA* with 0.2% casamino acids is similar to growth of the wild type with 0.05% casamino acids. This suggests that the mutant, although it can be complemented with casamino acids, it is still deficient compared to the wild type. Error bars represent standard deviation.
